# Low-grade sulfadoxine–pyrimethamine resistance in *Plasmodium falciparum* parasites from Lubango, Angola

**DOI:** 10.1186/s12936-016-1358-7

**Published:** 2016-06-07

**Authors:** Elsa P. S. Kaingona-Daniel, Larissa Rodrigues Gomes, Bianca E. Gama, Natália K. Almeida-de-Oliveira, Filomeno Fortes, Didier Ménard, Cláudio Tadeu Daniel-Ribeiro, Maria de Fátima Ferreira-da-Cruz

**Affiliations:** Laboratório de Pesquisa em Malária—Instituto Oswaldo Cruz, Fundação Oswaldo Cruz (Fiocruz), Rio de Janeiro, Brazil; Centro de Pesquisa, Diagnóstico e Treinamento em Malária (CPD-Mal) Fiocruz, Rio de Janeiro, Brazil; Hospital Central Dr. António Agostinho Neto, Lubango, Angola; Laboratory of Oncovirology, Instituto Nacional de Câncer, Rio de Janeiro, Brazil; Angolan National Malaria Control Programme, National Institute of Public Health, Luanda, Angola; Malaria Molecular Epidemiology Unit, Institut Pasteur in Cambodia, Phnom Penh, Cambodia; Health Progress and Investigation Network of the Portuguese-Speaking Countries Community (RIDESMal/CPLP), Lisbon, Portugal

**Keywords:** Malaria, *P. falciparum*, *pfdhfr*, *pfdhps*, Sulfadoxine, Pyrimethamine, Angola

## Abstract

**Background:**

Malaria is a major parasitic disease, affecting millions of people in endemic areas. *Plasmodium falciparum* parasites are responsible for the most severe cases and its resistance to anti-malarial drugs is notorious. This is a possible obstacle to the effectiveness of intermittent preventive treatment (IPT) based on sulfadoxine–pyrimethamine (SP) cures administrated to pregnant women (IPTp) during their pregnancy. As this intervention is recommended in Angola since 2006, it has assessed, in this country, the molecular profiles in *P. falciparumdhfr* and *dhps,* two polymorphic genes associated to pyrimethamine and sulfadoxine resistance, respectively.

**Methods:**

Blood samples from 52 falciparum patients were collected in Lubango, Angola and *pfdhfr* and *pfdhps* polymorphisms were analysed using nested-PCR and DNA sequencing.

**Results:**

In the *pfdhfr* gene, the 108**N** mutation was almost fixed (98 %), followed by 59**R** (63 %), 51**I** (46 %), 50**R** and 164**L** (2 %, respectively). No 16**V**/**S** mutations were found. The most common double mutant genotype was CN**RN** (59 + 108; 46 %), followed by C**I**C**N** (51 + 108; 29 %) whereas **IRN** (51 + 59 + 108; 15 %), CN**RN**VL (59 + 108 + 164; 2 %) and **RI**C**N** (50 + 51 + 108; 2 %) triple mutant genotypes were detected. Investigations of the *pfdhps* gene showed that the 437**G** mutation was the most prevalent (97 %). Only two and one samples disclosed the 540**E** (7 %) and the 436**A** (3 %), respectively. Single mutant S**G**KAA (437; 86 %) was higher than S**GE**AA (437 + 540; 7 %) or **AG**KAA (436 + 437; 3 %) double mutants genotypes. No polymorphism was detected at codons 581**G** and 613**T**/**S**. Combining *pfdhfr* and *pfdhps* alleles two triple mutant haplotypes (double mutant in *dhfr* and single mutant in *dhps*) were observed: the AC**I**C**N**VI/S**G**KAA in 14 (56 %) samples and the ACN**RN**VI/S**G**KAA in five (20 %) samples. One quadruple mutant haplotype was detected (AC**IRN**VI/S**G**KAA) in six (24 %) *P. falciparum* samples. No quintuple *pfdhfr*–*pfdhps* mutant was noted.

**Conclusion:**

*pfdhfr* and *pfdhps* gene mutations in isolates from Lubango are suggestive of a low-grade SP resistance and IPT for pregnant women and infant based on SP treatment could be effective. Routine molecular studies targeting polymorphism in these two genes need to be routinely conducted at country level.

## Background

*Plasmodium falciparum* malaria is the one of the major cause of morbidity and mortality in sub-Saharan Africa, including Angola, where three million clinical cases and 6000 deaths occurred in 2015. The deaths reported in Angola territory, accounting for 35 % in children under five years old and 25 % of the maternal deaths (Angolan National Malaria Control Programme, 2015).

In endemic malaria communities pregnant women and infants are more vulnerable to malaria episodes [1]. The World Health Organization strategy to protect mothers during their pregnancy and consequences in newborns such as low weight includes the implementation of intermittent preventive treatment in pregnant women (IPTp) [2]. This treatment was introduced in Angola in 2006, using the sulfadoxine–pyrimethamine (SP) at the second trimester of pregnancy. However, the emergence of *P. falciparum* resistances to both sulfadoxine and pyrimethamine can jeopardize these strategies.

Sulfadoxine–pyrimethamine resistant parasites are frequent in Southeast Asia and are becoming more and more prevalent in several East African countries [3]. SP acts by inhibiting the *P. falciparum* dihydrofolate reductase (*dhfr*) and dihydropteroate synthetase (*dhps*), two fundamental enzymes involved in the folate biosynthesis pathway [4]. Single nucleotide polymorphisms (SNPs) leading non-synonymous mutations in these two genes have been shown to be associated with pyrimethamine (*dhfr*) and sulfadoxine (*dhps)* resistances. The Angolan National Malaria Control Programme intends to introduce IPT in infants, as has already been done in other sub-Saharan Africa countries. To inform current policy makers when recommending the use of SP in IPT, *dhfr* and *dhps* molecular markers are used to differentiate high and low grade SP resistance areas and to define its geographical distribution [5].

Malaria is endemic throughout Angola, but the transmission patterns is heterogenous varying from intense transmission and hyperendemicity in northern, meso-endemic stable in the centre, to seasonal or epidemic unstable malaria in the southern part of the country [6]. No study for investigating *pfdhfr* and *pfdhps* mutations was performed in southern part of Angola where Lubango is located. The great majority of previous studies were conducted at the northern in Luanda, [7, 8]; Uige [9, 10], Cabinda, Kwanza Norte and Malanje [10], followed by those at the Central Angola in Benguela [11] and Huambo [10].

The objective of this study was to investigate the polymorphism at codons A16**V**/**S**, C50**R**, N51**I**, C59**R**, S108**N**, V140**L** and I164**L** in the *pfdhfr* gene and at codons S436**A**/**F**/**C**, A437**G**, K540**E**, A581**G** and A613**T**/**S** in the *pfdhps* gene, in order to provide baseline data regarding the proportion of *P. falciparumpfdhfr* and *pfdhps* mutations, before SP-IPTi introduction in Lubango, an Angolan malaria stable transmission region.

## Methods

### Study site, blood samples and DNA extraction

Blood samples were collected in 2011 from 52 patients presenting falciparum malaria at the Central Hospital in Lubango, located in Huila Province, South of Luanda, Angola capital. Lubango is located in southern Angola, with an area of 79,022 Km^2^, 1790 m above sea level, with an estimated population of 1,414,115. Malaria transmission occurs throughout the year, with peaks during rainy season, between March and May. Malaria is mesoendemic and *P. falciparum* is the predominant malaria species. Inclusion criteria comprised individuals with *P. falciparum* aging over 12 years and no evidence of complicated malaria. The Central Hospital is the main health facility in Lubango and it is a reference for malaria in the area. The malaria diagnosis was performed by thick blood smear and nested-PCR, as previous described [12]. All malaria cases were treated following the Angolan malaria treatment policy. After obtaining informed consent, venous blood collection was performed according to protocols approved by the National Institute of Public Health and the Ethical Committee of Angola and Fiocruz, Brazil (#372/07). The samples were cryopreserved and stored at −20 °C until DNA extraction. Genomic DNA was extracted from 1 mL whole blood using the QIAamp Midi columns (Qiagen), as described by the manufacturer.

### Nested polymerase chain reaction (PCR) and electrophoresis

*pfdhfr* and *pfdhps* genes were amplified by nested-PCR approach using two gene-specific primers (external and internal) as already reported [13]. The nested-PCRs were performed to detect the presence of mutations at codons: A16**V**/**S**, C50**R**, N51**I**, C59**R**, S108**N**, V140**L** and I164**L** in the *pfdhfr* gene and at codons S436**A**/**F**/**C**, A437**G**, K540**E**, A581**G** and A613**T**/**S** in the *pfdhps* gene. The choice of these codons was based on the pioneer study of Pearson and colleagues [13].

### DNA sequencing and SNP polymorphisms detection

After purification of the amplicons using the Wizard SV Gel and PCR Clean-Up System (Promega), PCR products were sequenced using Big Dye^®^ Terminator Cycle Sequencing Ready Reaction version 3.1 (Applied Biosystems) and ABI PRISM 3730 DNA Analyzer [14] at the Genomic Platform/PDTIS/Fiocruz. The sequences of the amplicons were aligned with the wild-type 3D7 strain for *pfdhfr* (GenBank accession number XM_001351443) and for *pfdhfr* (GenBank accession number Z30654). The presence of SNPs was confirmed by reading both the forward and the reverse strands using the free software Bioedit Sequence Alignment Editor Version 7.2.5. Parasites with mixed alleles (in which both wild-type and mutant alleles were present) were considered mutants for estimation of the prevalence of the SNPs. Haplotypes for drug resistance markers were reconstructed from the full sequence presenting an unambiguous single allele signal at all positions. Statistical significance of differences between the haplotypes was assessed using Fisher’s tests and a *p* value <0.05 was considered significant.

## Results

Among the 52 successfully sequenced samples for *dhfr*, 51 (98 %) showed non-synonymous mutations. The most frequent mutation was 108**N** (51/52; 98 %, 95 % CI) followed by 59**R** (33/52; 63 %, 95 % CI) and 51**I** (24/52; 46 %, 95 % CI). The 50**R** and 164**L** mutations were detected at the same frequencies (1/52; 2 %, 95 % CI) and no mutations were found at codons 16**V**/**S** and 140**L** (Table 1).

**Table 1:** *Plasmodium falciparum* *dhfr* and *dhps* mutated codons in parasites from Lubango, Angola

Gene	SNPs	Prevalence N (%)
*dhfr* (n = 52) 95 % CI	50**R**	1 (2)
	51**I**	24 (46)
	59**R**	33 (63)
	108**N**	51 (98)
	164**L**	1 (2)
*dhps (n* *=* *29)* 95 % CI	436**A**	1 (3)
	437**G**	28 (97)
	540**E**	2 (7)

Double mutants (51**I**/108**N**, 59**R**/108**N**) were observed in 39 isolates (76.5 %; 39/51) and triple mutants (51**I**/59**R**/108**N**, 50**R**/51**I**/108**N**, 59**R**/108**N**/164**L**) in ten (19.5 %; 10/51) *P. falciparum* samples. The 108**N** single mutant was found in only two (4 %; 2/51) isolates (Table 1). In samples presenting *dhfr* double mutation, the CN**RN** (59 + 108) allele was the most frequent (24/46 %) followed by C**I**C**N** (51 + 108) (15/29 %). This contrasts with the frequency of the *dhfr* triple mutant alleles which were detected at lower rates: **IRN** (51 + 59 + 108), CN**RN**V**L** (59 + 108 + 164) and **RI**C**N** (50 + 51 + 108) (8 (15 %), 1 (2 %) and 1 (2 %), respectively). The wild-type ACNCSVI was presented in only one isolate (Tables 2 and 3).

**Table 2:** Prevalence of *P. falciparum* *dhfr* and *dhps* mutants in parasites from Lubango, Angola

Gene	Mutation	SNPs	Prevalence N (%)
*dhfr n* = *51*	Single	S108**N**	2 (4)
	Double	N51**I/**S108**N**	15 (29)
		C59**R/**S108**N**	24 (47)
	Triple	N51**I**/C59**R/**S108**N**	8 (16)
		C50**R**/N51**I**/S108**N**	1 (2)
		C59**R**/S108**N**/I164**L**	1 (2)
*dhps n* = *28*	Single	A437**G**	25 (89)
	Double	A437**G**/K540**E**	2 (7)
		S436**A**/A437**G**	1 (4)

**Table 3:** Deduced haplotype profiles for *dhfr* and *dhps* *P. falciparum* parasites from Lubango, Angola

Gene	Haplotypes	N	%
*dhfr* (n = 52)	CN**RN**	24	46
	C**I**C**N**	15	29
	C**IRN**	8	15
	CNC**N**	2	4
	CN**RN**V**L**	1	2
	**RI**C**N**	1	2
	ACNCSVI	1	2
*dhps* (n = 29)	S**G**KAA	25	86
	S**GE**AA	2	7
	**AG**KAA	1	3
	SAKAA	1	3

Of the 52 samples tested, 29 (56 %) were successfully amplified for the *dhps* gene. Among them 28 (97 %) presented a mutation at codons S436**A**, A437**G** and K540**E**. The 437**G** mutant allele was highly prevalent (97 %), while the 540**E** (7 %) and 436**A** (3 %) were observed in lower frequencies. No 581**G** and 613**T**/**S** alleles were observed (Table 1). The frequency of the single mutant S**G**KAA (437) (25/86 %) was higher than double mutants S**GE**AA (437 + 540) (2/7 %) or **AG**KAA (436 + 437) (1/3 %) (p < 0.05). The *dhps* wild-type SAKAA was rare and observed in only one (3 %) *P. falciparum* sample (Tables 2 and 3).

Combining *pfdhfr* and *pfdhps* alleles, not all isolates had an interpretable haplotype (Table 4). The PCR amplification failure rate of the *dhps* gene in Lubango samples might be somehow attributed to primer mismatched due to unknown polymorphisms in target sequences, because in all the 52 samples amplification was achieved when primers strictly developed to amplify conserved sequences for diagnosis purposes were tested [12]. The number of samples herein considered was based on amplification of both PCRs just to avoid the identification of incomplete haplotypes.

**Table 4:** Combined molecular *dhfr* and *dhps* gene profiles in 25 samples of *P. falciparum* from Lubango, Angola

Genes	Haplotypes	Mutated	
*dhfr* + *dhps*		N/ %	Type
51I/108 N + 437G	AC**I**C**N**VI + S**G**KAA	14/56	Double and single
59R/108 N + 437G	ACN**RN**VI + S**G**KAA	5/20	Double and single
51I/59R/108 N + 437G	AC**IRN**VI + S**G**KAA	6/24	Triple and single

Two triple mutant haplotypes (double mutant in *dhfr* and single mutant in *dhps*) were observed: the AC**I**C**N**VI/S**G**KAA in 14 (56 %) samples and the ACN**RN**VI/S**G**KAA in five (20 %) samples. One quadruple mutant haplotype was detected (AC**IRN**VI/S**G**KAA) in six (24 %) *P. falciparum* samples (Table 4). The high-grade SP resistant haplotypes (quintuple, sextuple and septuple *pfdhfr/pfdhps*) [3] were not seen among these isolates.

## Discussion

At present, SP continues to be used in IPTp and IPTi strategies, which are likely to provide sub-optimal effect. Therefore, monitoring the spread of SP resistance using *dhfr* and *dhps* as molecular markers are strongly recommended [15]. This study was performed with *P. falciparum* samples from Lubango, an Angola region with unstable meso-endemic malaria transmission. The vast majority of tested isolates (98 %) presented at least one mutation in the gene encoding enzymes associated with pyrimethamine resistance.

High prevalence of 108**N** mutation (considered as the initial mutation occurring in *dhfr* multiple mutants) was detected, similar to observations already done in several Angolan regions [8, 10, 11] and in other African countries [16–26]. However, contrary to various Angolan localities, the 59**R** was the second more frequent *dhfr* mutation in Lubango, instead of the 51**I** [8–11]. In addition, it is worth to noting that the 164**L***pfdhfr* SNP was also detected for the first time in Angola. This mutation is rare in Africa [5] but its presence in association with 108**N** + 51**I** and/or 59**R** [27] as well as with the *dhfr* + *dhps* quintuple mutant [5], confers high levels of in vivo and in vitro resistance that could render SP totally ineffective. The 164**L** mutation was also combined to cycloguanil resistance [28] and is commonly found in *P. falciparum* samples from Southwest Asia [29, 30] and South America [31]. The possible reason for the detection of mutant 164**L** in Lubango (north of Angola) and not in other correlate Angolan studies (North and Central Angola) is, probably, due to the greater proximity of Lubango with Zambia, where the presence of quintuple mutants is greater than 50 % [3].

From previous reports, it was known that the triple African mutant (N51**I** + C59**R** + S108**N**) shares a common ancestry with resistant *dhfr* from Southeast Asia [32, 33]; this mutant emerged in Asia and was introduced later in Africa, including Angola [19, 20, 33–36]. With respect to *dhfr* mutation 50**R,** another non-frequent *dhfr* mutation in Africa, it was also identified in this study. This low-grade pyrimethamine resistant mutant [27] was first described as specific of South American isolates [27] but, besides South America, it has already been described in Africa in isolates from Kenya [35], Central African Republic [37] and Angola [8].

In relation to *dhps* gene, the 437**G** mutation was the most common, reaching almost fixation, similarly to other Angolan localities [8, 10, 11] and other African countries [16, 20, 23, 26], especially in western and Central Africa.

The *dhps* 540**E** mutation, less commonly observed in western Africa, was also identified in this study, confirming previous data from Luanda [7, 8], Cabinda, Huige, Kwanza Norte, Malange and Huambo [10]. However, the spatial distribution of this allele in Angolan provinces seems heterogeneous and even in the same province its frequency can vary overtime [9, 10]. Thus, screening for *dhps* 540**E**, a surrogate for the quintuple mutant, is a priority in West Africa where it is comparatively rare [3].

The combination of 437**G** with the triple mutant 51**I** + 59**R** + 108**N** which is considered to be associated with SP treatment failure [38–40], was detected in 33 % of Lubango samples. However, in vitro studies showed that the single 437**G** mutant has a lesser degree of tolerance to sulfadoxine than the 437**G** + 540**E** double mutant. Thus, triple mutant 51**I** + 59**R** + 108**N** associated with single 437**G** mutant could have a less detrimental effect on SP-IPT than the associated 437**G** + 540**E** mutations—the sustained protective efficacy of SP-IPTi in western and Central Africa reinforce these points [41].

Despite the presence of parasites with triple *dhfr* mutant at positions 51**I**, 59**R** and 108**N** (15 %) and double *dhps* mutant at positions 437**G** + 540**E** (7 %), the quintuple mutant (51**I** + 59**R** + 108**N** + 437**G** + 540**E**), considered a significant predictor of SP treatment failure [42] was not detected in the studied isolates from Lubango. To date, this quintuple mutant was not observed in central and northern areas of Angola [7–11]. Besides Lubango, similar low frequencies of double 437**G** + 540**E***pfdhps* mutant were described in Huambo (12.5 %) [10], Benguela (8 %) [10], Cabinda (5.3 %) [10], Malanje (3.1 %) [10], Luanda (3 %) [8], Kwanza Norte (2.1 %) [10] and Uige (1.9 %) [10], contrasting with the higher frequencies of this mutant detected in Eastern Africa: Kenya (86 %), Tanzania (90 %), Malawi (95 %) and Uganda (95 %). With the exception of Malanje (3.9 %), the frequencies of the triple 51**I** +59**R** + 108**N***dhfr* mutant in Angolan provinces, including Lubango, were higher than the double 437**G** + 540**E***pfdhps* mutant but, East African countries presented higher prevalence rates [43].

These findings are expected because East African countries presented a high rate of SP treatment failure of falciparum malaria together with the increased presence of the quintuple mutant considered the most specific molecular marker for SP [44, 45].

Besides quintuple mutants, the concept of “super resistant genotypes” is emerging which further raise the threshold of drug tolerance in parasites comprising additional mutations in combination with N51**I** + C59**R** + S108**N** and A437**G** + K540**E** [3]. The additional mutations are *dhfr* I164**L** (found in one sample; 2 %) and *dhps* A581**G** and A613**T**/**S**, which were not found in this study. Although, these mutations are rare, their impact on SP-IPTi or SP-IPTp efficacy is considered highly detrimental [5]. When the quadruple mutant N51**I** + C59**R** + S108**N** + A437**G** occur in the absence of 540**E**, these parasite populations are classified as partially resistant genotypes, and its resistance levels are not expected to be as high [3]. The efficacy of IPTp in western Africa, where N51**I** + C59**R** + S108**N** and A437**G** exist in combination with 581**G** but not with 540**E** supports the view that these parasites should be regarded differently [3]. In this case, molecular markers of super resistance may be an important warning about failing efficacy of IPTp.

Many studies suggested that IPTp with SP remains effective, or at least it is not associated with harm, in areas with high prevalence of *P. falciparum* quintuple mutant parasites [46, 47]. There is not enough evidence yet to establish a threshold prevalence of the classical *dhfr* + *dfps* quintuple mutant, or even of the *dhps* 581, *dhps* 540 or *dhfr* 164 point mutations above which there is a clear loss of IPTp-SP effectiveness. Therefore, the significance of the additional mutation at codon 540 needs further investigation.

## Conclusion

In view of the data reported here and elsewhere, it was conclude that, although the number of samples available for the present study was limited, *pfdhfr* and *pfdhps* gene mutations in isolates from Lubango are suggestive of a low-grade SP resistance and their presences highlight the importance of molecular markers screening to monitor the evolution of *P. falciparum* SP resistance in Angola.

## References

[1] Tutu EO, Lawson B, Browne E (2011). The effectiveness and perception of the use of sulphadoxine-pyrimethamine in intermittent preventive treatment of malaria in pregnancy programme in Offinso district of Ashanti region, Ghana. Malar J..

[2] WHO. A strategic framework for malaria prevention and control during pregnancy in the African region, World Health Organization. 2004.

[3] Naidoo I, Roper C (2013). Mapping ‘partially resistant’, ‘fully resistant’ and ‘super resistant’ malaria. Trends Parasitol.

[4] Brooks DR, Wang P, Read M, Watkins WM, Sims PF, Hyde JE (1994). Sequence variation of the hydroxymethyldihydropterin pyrophosphokinase: dihydropteroate synthase gene in lines of the human malaria parasite, *Plasmodium falciparum*, with differing resistance to sulfadoxine. Eur J Biochem.

[5] Venkatesan M, Alifrangis M, Roper C, Plowe CV (2013). Monitoring antifolate resistance in intermittent preventive therapy for malaria. Trends Parasitol.

[6] Angola malaria indicator survey 2011. final report. http://dhsprogram.com/pubs/pdf/MIS11/MIS11.pdf.

[7] Figueiredo P, Benchimol C, Lopes D, Bernardino L, Rosario VE, Varandas L (2008). Prevalence of pfmdr1, pfcrt, pfdhfr and pfdhps mutations associated with drug resistance, in Luanda, Angola. Malar J.

[8] Gama BE, Pereira-Carvalho GA, Lutucuta Kosi FJ, Almeida de Oliveira NK, Fortes F, Rosenthal PJ (2011). Molecular markers of antifolate resistance in *Plasmodium falciparum* isolates from Luanda, Angola. Malar J..

[9] Menegon M, Pearce RJ, Inojosa WO, Pisani V, Abel PM, Matondo A, Bisoffi Z, Majori G (2009). Monitoring for multidrug-resistant *Plasmodium falciparum* isolates and analysis of pyrimethamine resistance evolution in Uige province, Angola. Trop Med Int Health.

[10] Fortes F, Dimbu R, Figueiredo P, Neto Z, do Rosário VE, Lopes D (2011). Evaluation of prevalences of pfdhfr and pfdhps mutations in Angola. Malar J..

[11] Ngane FV, Allico Djaman J, Culeux C, Piette N, Carnevale P, Besnard P (2015). Molecular epidemiology of drug-resistant *Plasmodium falciparum* in Benguela province, Angola. Malar J..

[12] Zalis MG, Ferreira-da-Cruz MF, Balthazar-Guedes HC, Banic DM, Alecrim W, Souza JM (1996). Malaria diagnosis: standardization of a polymerase chain reaction for the detection of *Plasmodium falciparum* parasites in individuals with low-grade parasitemia. Parasitol Res.

[13] Pearce RJ, Drakeley C, Chandramohan D, Mosha F, Roper C (2003). Molecular determination of point mutation haplotypes in the dihydrofolate reductase and dihydropteroato synthase of *Plasmodium falciparum* in three districts of northern Tanzania. Antimicrob Agents Chemother.

[14] Otto TD, Vasconcellos EA, Gomes LH, Moreira AS, Degrave WM, Mendonça-Lima L (2008). ChromaPipe: a pipeline for analysis, quality control and management for a DNA sequencing facility. Genet Mol Res.

[15] Alifrangis M, Nag S, Schousboe ML, Ishengoma D, Lusingu J, Pota H (2014). Independent origin of *Plasmodium falciparum* antifolate super-resistance, Uganda, Tanzania and Ethiopia. Emerg Infect Dis.

[16] Gebru-Woldearegai T, Hailu A, Grobusch MP, Kun JF (2005). Molecular surveillance of mutations in dihydrofolate reductase and dihydropteroate synthase genes of *Plasmodium falciparum* in Ethiopia. Am J Trop Med Hyg.

[17] Schunk M, Kumma WP, Miranda IB, Osman ME, Roewer S, Alano A, Löscher T (2006). High prevalence of drug-resistance mutations in *Plasmodium falciparum* and *Plasmodium vivax* in southern Ethiopia. Malar J..

[18] Nkhoma S, Molyneux M, Ward S (2007). Molecular surveillance for drug-resistant *Plasmodium falciparum* malaria in Malawi. Acta Trop.

[19] Certain LK, Briceno M, Kiara SM (2008). Characteristics of *Plasmodium falciparum* dhfr haplotypes that confer pyrimethamine resistance, Kilifi, Kenya, 1987–2006. J Infect Dis.

[20] Lynch C, Pearce R, Pota H (2008). Emergence of a *dhfr* mutation conferring high-level drug resistance in *Plasmodium falciparum* populations from Southwest Uganda. J Infect Dis.

[21] Zhong D, Afrane Y, Githeko A, Cui L, Menge DM, Yan G (2008). Molecular epidemiology of drug-resistant malaria in western Kenya highlands. BMC Infect Dis.

[22] Alifrangis M, Lusingu JP, Mmbando B, Dalgaard MB, Vestergaard LS, Ishengoma D (2009). Five-year surveillance of molecular markers of *Plasmodium falciparum* antimalarial drug resistance in Korogwe District, Tanzania: accumulation of the 581G mutation in the *P. falciparum* dihydropteroate synthase gene. Am J Trop Med Hyg.

[23] Bonizzoni M, Afrane Y, Baliraine FN, Amenya DA, Githeko AK, Yan G (2009). Genetic structure of *Plasmodium falciparum* populations between lowland and highland sites and antimalarial drug resistance in Western Kenya. Infect Genet Evol.

[24] Oesterholt MJ, Alifrangis M, Sutherland CJ, Omar SA, Sawa P, Howitt C (2009). Submicroscopic gametocytes and the transmission of antifolate-resistant *Plasmodium falciparum* in Western Kenya. PLoS One.

[25] Karema C, Imwong M, Fanello CI, Stepniewska K, Uwimana A, Nakeesathit S (2010). Molecular correlates of high-level antifolate resistance in Rwandan children with *Plasmodium falciparum* malaria. Antimicrob Agents Chemother.

[26] Salgueiro P, Vicente JL, Ferreira C, Teófilo V, Galvão A, do Rosário VE (2010). Tracing the origins and signatures of selection of antifolate resistance in island populations of *Plasmodium falciparum*. BMC Infect Dis.

[27] Cortese JF, Caraballo A, Contreras CE, Plowe CV (2002). Origin and dissemination of *Plasmodium falciparum* drug-resistance mutations in South America. J Infect Dis.

[28] Basco LK, Pécoulas PE, Wilson CM, Le Bras J, Mazabrald A (1995). Point mutations in the dihydrofolate reductase-thymidylate synthase gene and pyrimethamine and cycloguanil resistance in *Plasmodium falciparum*. Mol Biochem Parasitol.

[29] Foote SJ, Gálatas D, Cowman AF (1990). Amino acids in the dihydrofolate reductase-thymidylate synthase gene of *Plasmodium falciparum* involved in cycloguanil resistance differ from those involved in pyrimethamine resistance. Proc Nat Acad Sci USA.

[30] Peterson DS, Milhous WK, Wellems TE (1990). Molecular basis of differential resistance to cycloguanil and pyrimethamine in *Plasmodium falciparum* malaria. Proc Nat Acad Sci USA.

[31] Vasconcelos KF, Plowe CV, Fontes CJ, Kyle D, Wirth DF, Pereira da Silva LH (2000). Mutations in *Plasmodium falciparum* dihydrofolate reductase and dihydropteroate synthase of isolates from the Amazon region of Brazil. Mem Inst Oswaldo Cruz.

[32] Roper C, Pearce R, Nair S, Sharp B, Nosten F, Anderson T (2004). Intercontinental spread of pyrimethamine-resistant malaria. Science.

[33] Maiga O, Djimde AA, Hubert V (2007). A shared Asian origin of the triple-mutant dhfr allele in *Plasmodium falciparum* from sites across Africa. J Infect Dis.

[34] Roper C, Pearce R, Bredenkamp B (2003). Antifolate antimalarial resistance in southeast Africa. Lancet.

[35] McCollum AM, Poe AC, Hamel M (2006). Antifolate resistance in *Plasmodium falciparum*: multiple origins and identification of novel dhfr alleles. J Infect Dis.

[36] Ndiaye D, Daily JP, Sarr O (2006). Defining the origin of *Plasmodium falciparum* resistant dhfr isolates in Senegal. Acta Trop.

[37] Menard D, Djalle D, Yapou F, Manirakiza A, Talarmin A (2006). Frequency distribution of antimalarial drug-resistant alleles among isolates of *Plasmodium falciparum* in Bangi, Central African Republic. Am J Trop Med Hyg.

[38] Mockenhaupt FP, Teun BJ, Eggelte TA, Schreiber J, Ehrhardt S, Wassilew N, Otchwemah RN, Sauerwein RW, Bienzle U (2005). *Plasmodium falciparum* dhfr but not dhps mutations associated with sulphadoxine-pyrimethamine treatment failure and gametocyte carriage in Northern Ghana. Trop Med Int Health..

[39] Kun JF, Lehman LG, Lell B, Schimidt-Ott R, Kremsner PG (1999). Low-dose treatment with sulfadoxine–pyrimethamine combinations select for drug-resistant *Plasmodium falciparum* strains. Antimicrob Agents Chemother.

[40] Ndounga M, Tahar R, Basco LK, Casimiro PN, Malonga DA, Ntoumi F (2007). Therapeutic efficacy of sulfadoxine–pyrimethamine and the prevalence of molecular markers of resistance in under 5-year olds in Brazzaville. Congo. Trop Med Int Health..

[41] Griffin JT, Cairns M, Ghani AC, Roper C, Schellenberg D, Carneiro I (2010). Protective efficacy of intermittent preventive treatment of malaria in infants (IPTi) using sulfadoxine–pyrimethamine and parasite resistance. PLoS One.

[42] Picot S, Olliaro, de Monbrinson F, Bienvenu AL, Price RN, Ringwald P (2009). A systematic review and meta-analysis of evidence for correlation between molecular markers of parasite resistance and treatment outcome in falciparum malaria. Malar J..

[43] Sridaran S, McClintock SK, Syphard LM, Herman KM, Barnwell JW, Udhayakumar V (2010). Anti-folate drug resistance in Africa: meta-analysis of reported dihydrofolate reductase (dhfr) and dihydropteroate synthase (dhps) mutant genotype frequencies in African *Plasmodium falciparum* parasite populations. Malar J..

[44] Omar SA, Adagu IS, Gump DW, Ndaru NP, Warhurst DC (2001). *Plasmodium falciparum* in Kenya: high prevalence of drug-resistance-associated polymorphisms in hospital admissions with severe malaria in an epidemic area. Ann Trop Med Parasitol.

[45] Staedke SG, Sendagire H, Lamola S, Kamya MR, Dorsey G, Rosenthal PJ (2004). Relationship between age, molecular markers, and response to sulphadoxine-pyrimethamine treatment in Kampala. Uganda. Trop Med Int Health.

[46] WHO. Policy recommendation: intermittent preventive treatment of malaria in pregnancy using sulfadoxine–pyrimethamine (IPTp-SP). Geneva, World Health Organization, 2012.

[47] Gutman J, Kalilani L, Taylor S, Zhou Z, Wiegand RE, Thwai KL (2015). The A581G mutation in the gene encoding *Plasmodium falciparum* dihydropteroate synthetase reduces the effectiveness of sulfadoxine–pyrimethamine preventive therapy in Malawian pregnant women. J Infect Dis.

